# Structure Formation of Ultrathin PEO Films at Solid Interfaces—Complex Pattern Formation by Dewetting and Crystallization

**DOI:** 10.3390/ijms14023254

**Published:** 2013-02-05

**Authors:** Hans-Georg Braun, Evelyn Meyer

**Affiliations:** Max Bergmann Center of Biomaterials, Leibniz Institute of Polymer Research Dresden, Hohe Strasse 6, D-01069 Dresden, Germany

**Keywords:** dewetting, DLA, PEO, PEG, thin film, crystallization

## Abstract

The direct contact of ultrathin polymer films with a solid substrate may result in thin film rupture caused by dewetting. With crystallisable polymers such as polyethyleneoxide (PEO), molecular self-assembly into partial ordered lamella structures is studied as an additional source of pattern formation. Morphological features in ultrathin PEO films (thickness < 10 nm) result from an interplay between dewetting patterns and diffusion limited growth pattern of ordered lamella growing within the dewetting areas. Besides structure formation of hydrophilic PEO molecules, n-alkylterminated (hydrophobic) PEO oligomers are investigated with respect to self-organization in ultrathin films. Morphological features characteristic for pure PEO are not changed by the presence of the n-alkylgroups.

## 1. Introduction

**Figure 1:**
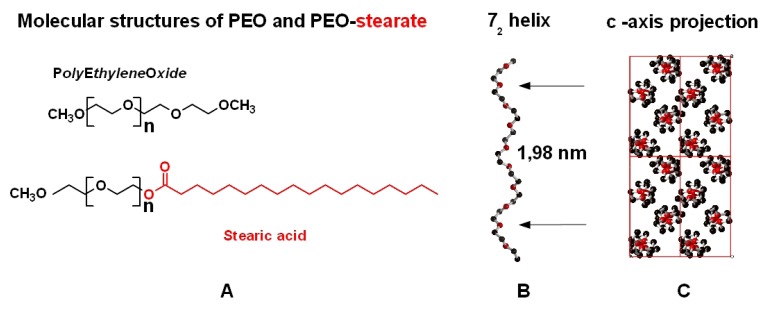
Molecular structure of PEO and PEO-stearate (**A**); helical chain conformation (**B**) and chain packing (**C**) of PEO.

Although the linear molecular structure of polyethyleneoxide (PEO) is very simple (see Figure 1A) it offers a number of important physicochemical properties. Perhaps the most important property is the ability of PEO layers to avoid non-specific protein adsorption on surfaces [1]. This behavior is generally seen as a consequence of the hydrogen bonding between ethyleneoxide (EO) segments and surrounding water molecules which induces local order at the molecular interface [2]. The high affinity of EO segments to water molecules is frequently used to modify hydrophobic molecules or macromolecules such as polythiophene [3] to become water soluble. In self-assembled monolayers of PEO entities it has been demonstrated that the molecular conformation in the ordered PEO phase either refers to a planar zigzag conformation or to a 7_2_ helical conformation [4]. 7_2_ helical [5] (Figure 1B,C) and planar zigzag conformation [6] were studied in detail by X-ray structure analysis and were identified to represent the 2 modifications realized in bulk crystallized PEO. It has been discussed that the molecular conformation controls the interfacial water layer [7] and therefore the protein adsorption [1]. Recent experiments [7] indicate the importance of local interfacial water structures for protein adsorption on surface grafted PEO thin films. Another observation related to the high affinity between PEO segments and water molecules was observed in PEO microdroplets prepared from organic solvents such as chloroform [8]. After evaporation of the organic solvent the residual PEO phase immediately becomes hydrated. Due to the high specific surface of the microdroplets water molecules immediately interact with PEO and keep the droplet in a liquefied state without crystallization or solidification.

The crystallization of PEO in ultrathin layers in a highly branched lamella structure has been extensively experimentally studied and theoretically described by Sommer and Reiter [9–11]. The influence of surface confinements and surface defects on nucleation and on growth patterns of PEO lamella has been clearly demonstrated [12]. Studies on ultrathin melt crystallized PEO films clearly indicate that although the lamella are highly branched, they represent at least extended 2-dimensional ordered structures as revealed by recent electron diffraction experiments [13].

Atomic force microscopy (AFM) studies on crystalline morphologies grown at various supercooling of molten ultrathin PEO films showed morphological transitions from regular square shaped PEO lamella crystals observed at low supercooling to highly branched lamella structures growing at high supercooling [14]. An interesting question to be discussed is the influence of aliphatic (hydrophobic) end-group modification on the self-organization of PEO in ultrathin films.

Esterification of OH end-groups of PEO oligomers with long chain n-alkyl units like stearic acid is used to prepare non-ionic surfactants with PEO as the hydrophilic and the n-alkyl groups as hydrophobic structural entities (Figure 1A). Depending on the average molecular weight of PEO segments attached to the stearate group, these molecules are used as non-ionic emulsifiers or as gelator in pharmaceutical cream formulations. While with short PEO chains (for example 9 PEO units in *Cremophor*^®^ S9) the self-assembly process in the presence of aqueous solutions favors a double layer formation of the hydrophobic alkyl groups, increasing the molecular weight of PEO units esterified with the stearic acid ( *Myrj*^®^ 59 ) does not indicate assembly of alkyl chains. X-ray diffractograms of such materials provide only evidence for crystallization of PEO segments [15].

Inspired by the structural variability of PEO, this article addresses the particular self-organization of PEO and stearate terminated PEO molecules in ultrathin films. Strong emphasis will be laid on the influence of dewetting structures on the morphology of self-assembled unilamellar entities.

## 2. Results and Discussion

### 2.1. Dewetting and Crystallization in Ultrathin PEO Films

**Figure 2:**
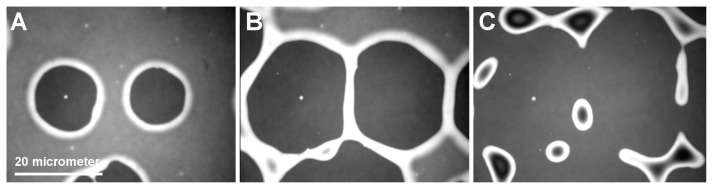
Successive morphological changes during dewetting indicating hole formation (**A**), growth (**B**) and rupture into droplets (**C**), scale bar refers to all figures.

**Figure 3:**
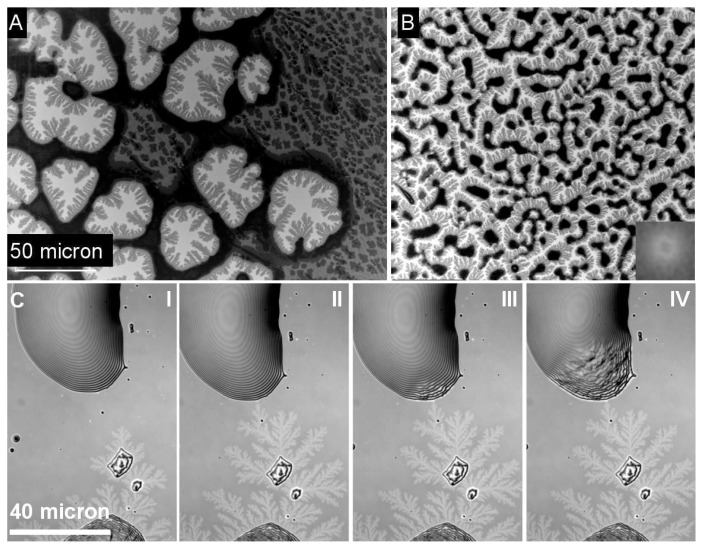
Heterogeneous (**A**) or spinodal dewetting (**B**) morphologies (SEM micrographs) The inset in Figure 3B refers to the Fast Fourier Transform (FFT) Calculation of the structure typical for spinodal dewetting. Dendritic growth in ultrathin layers between individual PEO droplets (C I-IV). (Contrast enhanced light microscopic images), identical scale bar for A, B and C I-IV.

In thin film preparation, dewetting is often regarded as an undesired effect. However the local control of dewetting by chemical [18] or topographical surface patterning can also be used as a thin film patterning microtechnology [19]. It is generally accepted that dewetting of thin polymer films follows either a heterogeneous process [20,21] or a spinodal type scenario [21,22]. Heterogeneous dewetting starts with the formation of holes at defect sites (Figure 2A). With progressive dewetting, holes grow until they form an extended network (Figure 2B and Figure 3A). The polymer bridges separating the holes become smaller until they decompose into lines of individual droplets according to a Rayleigh instability [23] (Figure 2C).In spinodal dewetting, dewetted and wetted areas form a bi-continuous structure. Patterns are similar to the morphology observed for two phase structure in spinodal decomposition of binary mixtures [24]. In spinodal dewetting, the thickness profile along an arbitrary straight line can be described by a single periodic (sinusoidal) function. In general the particular bi-continuous structure in spinodal processes is recognized and characterized by Fourier transform of the imaged structure as has been done for the characteristic dewetting structure of the thin PEO films imaged in Figure 3B.

It is worth to notice that thin film stability and therefore the critical thickness for the onset of the dewetting process is **not only** related to the surface chemistry but also dependent on the layer structure in multilayer substrates like in oxidized silicon wafers [25]. In particular, the oxide layer thickness between pure silicon and the film provides an essential contribution to the effective Hamacker constant to be considered in the calculation of long range interactions between substrate and film. We observed a significant decrease in PEO film stability with increasing oxide layers thickness. It is mostly assumed that dewetted areas are completely free of residual film and should consequently represent substrate surface properties. In the system under discussion, this is actually not true. In reality, the dewetted areas are still coated with an amorphous film of *ca.* 3 nm thickness [12]. Layer thickness measurement was only possible by ellipsometry because the AFM tip triggered the nucleation of the amorphous film. This amorphous PEO layer can be transformed into dendritic lamella polymer crystals, which reflect the diffusion limited aggregation growth process (DLA) [9]. The crystallization starts from the rims of the dewetted structures and propagates through the amorphous film. From Figure 3A and similar, we calculated that inside the holes, the ratio of surface area covered by crystalline PEO lamella to the overall surface area of the dewetted holes is almost constant and is equal to 0.42. The constant ratio observed for different dewetting structures provides strong evidence that the material does not diffuse from the rims but that it has already been present as residual layer after dewetting. If we assume a constant volume scenario for the transformation of amorphous PEO inside the hole into ordered lamella, we calculate the ratio of height of the crystalline lamella to amorphous film to be 1/0.42 = 2.38. Consequently, an amorphous PEO film of 3 nm thickness should provide uniform lamella of 2.38 × 3 nm (*ca.* 7 nm), which has been experimentally confirmed by AFM measurement.

As already demonstrated in Figure 2C, the final state of dewetting results in a field of supposed isolated liquid droplets (Figure 3C I-IV). Although generally not visible in a reflective light microscope setup, the droplets are surrounded by an ultrathin PEO layer already mentioned. If by external forces such as contact with an AFM tip [12] the crystallization in the ultrathin film is nucleated, the dendrites become visible in a modified reflection microscopy setup first published by Riegler *et al*. [16]. The method described by these authors is based on contrast enhancement from small thickness variations (nm size) of the sample imaged in an ordinary reflective light microscope. The only experimental requirement in the optical setup is the use of a reflecting multilayer substrate with well-defined layer thickness and dielectric constant and a monochromatic light source. Applying this technique, dendritic growth structures that appear in the amorphous film after nucleation can be visualized as shown in figure (ref Figure). If one of the isolated droplets starts to crystallize, it starts to nucleate the dendritic growth inside the ultrathin amorphous film between isolated droplets. The dendrites propagate the ultrathin film, and as soon as the growth front touches a liquid PEO droplet (see Figure 3C III-IV), crystallization inside the metastable drop occurs. This again triggers nucleation in the amorphous film surrounding the droplet and the overall crystallization in the film appears like a chain reaction.

### 2.2. Structure Formation of N-alkyl Terminated PEO

In a series of recent papers, detailed studies were published on the morphological appearance of short chain PEO lamella crystals grown at various undercooling within thin melt films. These included transmission electron microscopy (TEM) electron diffraction studies concluding on the relationship between growth habit and crystallographic structure, in particular chain orientation and packing.

**Figure 4:**
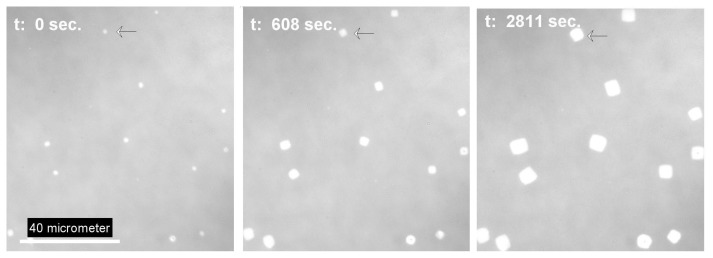
Crystal growth at isothermal crystallization of PEO-stearate at low supercooling (49 °C), scale bar refers to all figures.

**Figure 5:**
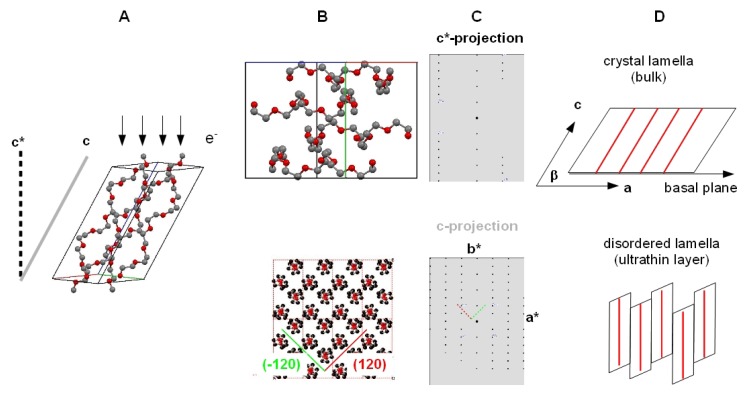
Molecular packing of PEO in the elementary cell and orientation of incident electron beam (**A**); Projection of molecules along the c star and c axes (**B**) and corresponding zone axis diffractions patterns (**C**); Molecular (dis)-order of chains within the lamella (**D**).

In our studies, we like to compare the morphological appearance thus described with that observed on the crystallization of stearate terminated PEO oligomers [15] that we used in our experiments. During isothermal crystallization conditions, the growth of well-defined, almost square-shaped lamella crystals is observed (see Figure 4). The growth pattern of these crystals is almost similar to that observed for pure PEO samples under similar crystallization conditions [26]. Lotz *et al*. [13] demonstrated that in pure PEO, the nearly square-shaped growth facets correspond to (1̄20) and (120) lattice planes, which intersect with an angle of 89.63°. The zone axis of these lattice planes, which is similar to the direction of the polymer chains, is the 00l or crystallographic c-axes of the monoclinic elementary cell. For this orientation, the expected TEM zone axis diffraction pattern corresponds to the c-projection indicated in Figure 5, which means that the polymer chains are oriented perpendicular to the substrate. The identical morphological features of the stearate terminated PEO and pure PEO suggests a similar packing model. We like to point out that in a monoclinic elementary cell in which the (001) lattice plane is oriented parallel to the basal plane of the crystal lamella, a different zone axis diffraction pattern would appear (Figure 5). If as observed PEO chains are found perpendicular to the surface, the lamella basal planes would have to be inclined to the by an angle similar to the tilt angle of PEO segments with respect to the (00l) plane, which is very much unlikely. Concerning ultrathin films, all experimental observations published until now [13] are compatible with a model assuming that the PEO segments are perpendicular to the substrate surface, and we suggest that that chains have a translational disorder along the chain axis. Such a disorder would not affect the molecular packing as seen by the corresponding TEM zone axis diffraction pattern along the [001] axis. AFM measurements on melt crystallized lamella in thin PEO-stearate films strongly support the proposed model.

**Figure 6:**
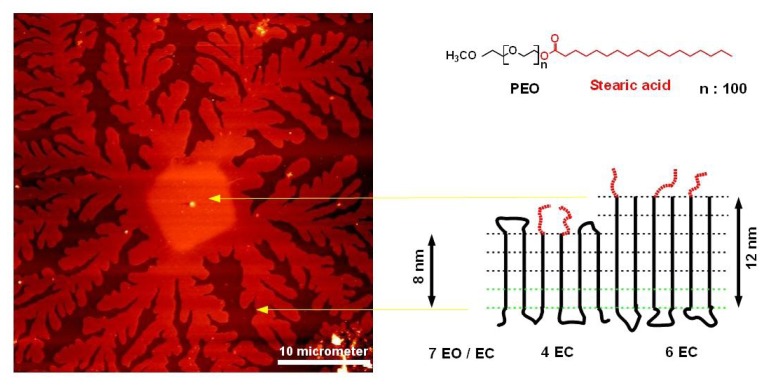
AFM image of PEO-stearate lamella and scheme of molecular packing arrangement inside lamella structures. Experimental data from AFM height measurement are indicated in the scheme. (EC = elementary cell, EO = ethyleneoxide unit).

Figure 6 shows an AFM image of a PEO lamella crystal, which started to grow under isothermal conditions at low undercooling (49 °C) which appears in the square-shaped growth morphology as discussed before. Rapid cooling of the crystal in the later stage of the crystallization caused dendritic lamella structures, which reflects the non-equilibrium aggregation (DLA) process. Based on lamella crystal height measurements both in the central square shape area and in the branched region, a folding process schemed in Figure 6 is compatible with our experimental data. A tilt of the chain axis would reduce the lamella heights by 18%.

**Figure 7:**
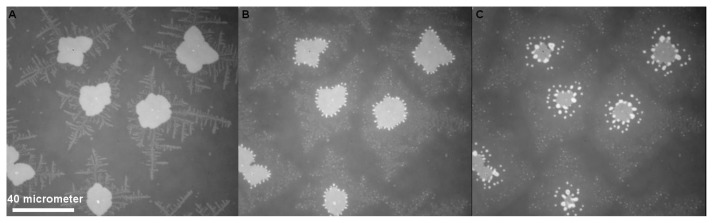
Different wetting and dissolution behavior on PEO-stearate lamella during progressive water condensation at the dew point, scale bar refers to all figures.

A consequence of the difference in the chain folding scheme between the central and the branched parts of the lamella is the change in the density of hydrophobic end-groups to be expected on the surface of the different morphologies. A statistical distribution of hydrophobic end-groups on both sides of the lamella would give a probability of 0.25 for the thicker central part, respectively 0.17 for the thinner dendritic part. In order to prove a possible difference on the surface properties of the different crystal areas, we investigated the condensation behavior with respect to water at the dew point. As demonstrated by Figure 7, the behavior of the thick, more hydrophobic parts with respect to condensed water is clearly different compared with the branched, less hydrophobic parts. The branches do not indicate any droplet formation, which refers to a high wettability. The suspected more hydrophobic thicker parts clearly become decorated by droplets, which are most probably condensed water droplets. The shape of the droplets indicates a large contact angle. Furthermore, the thinner branches become already dissolved while the more hydrophobic parts seem to be protected from the dissolution process, although one should keep in mind that the thinner lamellas have a lower dissolution energy compared with the thicker ones.

## 3. Experimental Section

### 3.1. Materials

Polyethyleneoxide (*M_w_* 2000 g/mol, Rapp Polymers Tuebingen) and polyethyleneoxide (*M_w_* 4500 g/mol, *P_n_*_(_*_PEO_*_)_ = 100 Sigma Aldrich) esterified with stearic acid were used. For film formation, solutions of PEO or PEO-stearate in chloroform (Merck, analytical grade) were prepared (typical concentration 0.15% w/w). As substrates, silicon wafers with either a native oxide layer or with a thermal oxidized oxide layer of 300 nm thickness were used (SiMat Silicon Materials, Landsberg Lech).

### 3.2. Methods

Ultrathin films of PEO, respectively PEO-stearate, on cleaned silica wafers were prepared by dip-coating substrates from PEO or PEO-Stearate solutions at constant speed (*v* = 6 mm/min) using a commercial film-lift (Riegler und Kirstein GmbH, Berlin). Prior to coating, wafers were ultrasonicated in absolute alcohol, rinsed and dried under a nitrogen flow. For crystallization experiments, dip-coated films were molten at ambient temperatures and crystallized under temperature control of a heating stage (LINKAM THMS 600). Non-isothermal growth was observed under cooling rates of 1 °C/min. Thin film lamella growth was observed by reflective light microscopy (Zeiss Axiskop) using monochromatic illumination (*λ_max_* 435 nm) from a LED source (CoolLED) attached to the microscope. For contrast enhancement, the use of silicon wafer substrates with an oxide layer of 300 nm is essential [16]. Imaging of ultrathin crystalline layers was recorded with a 14-bit camera (Spot). Image analysis was done with the ImageJ [17] software. For wetting experiments, samples were cooled below the dew point in order to create water droplets by condensation from the atmosphere. Cooling was realized by on a self-made cooling stage based on a Peltier element attached to an ordinary lab power supply. For light microscopic inspection of thin film crystal growth, samples were prepared on appropriate oxide layer and irradiated by monochromatic light obtained from an LED source (CoolLED) without further monochromatization.

Scanning electron microscopy (SEM) inspection of samples was done in a Zeiss Gemini SEM operated at low voltages (0.300 keV< *E*_0_ <1 keV) in order to achieve favorable conditions for surface and thin film imaging at low penetration depth of electrons and to avoid charging while samples were imaged as prepared without additional metal or carbon coating.

Quantification of lamella height was done by AFM measurement using an AFM setup attached to a reflective light microscope (SIS Ultraobjective). This setup allows a pre-selection of ultrathin lamella in the light microscope.

Zone axis diffraction patterns of simulated electron diffraction data were calculated from atom coordinates published in literature [5] using a commercial software (CrystalMaker and CrystalDiffract, Crystal Maker Software Ltd., http://www.crystalmaker.com/).

## 4. Conclusions

We investigated the dewetting behavior and crystallization in ultrathin PEO films. Dewetted areas are covered with an amorphous film, which can be nucleated to start crystallization into highly branched PEO lamella by a diffusion limited aggregation process. The propagating crystallization front could be imaged by a modified reflective light microscopic method.

Modification of oligoethyleneoxides by stearic acid generates similar dendritic lamella to those observed for pure PEO of the same molecular weight. The crystallization of these compounds is obviously characterized by the self-organization of PEO segments and not by self-organization of the long chain alkyl groups into close packed paraffin membranes.
